# Distal Renal Tubular Acidosis in an Iranian Patient with Hereditary Spherocytosis

**DOI:** 10.52547/ibj.25.5.359

**Published:** 2021-08-28

**Authors:** Zahra Shahab-Movahed, Ahmad Majd, Elham Siasi Torbati, Sirous Zeinali

**Affiliations:** 1 Department of Cellular and Molecular Biology, Faculty of Science, North Tehran Branch of Islamic Azad University, Tehran, Iran;; 2 Department of Genetic, Faculty of Science, North Tehran Branch of Islamic Azad University, Tehran, Iran;; 3 Department of Medical Molecular, Biotechnology Research Center, Pasteur Institute of Iran, Tehran, Iran;; 4 Kawsar Human Genetic Research Center, Tehran, Iran

**Keywords:** Erythrocyte membrane protein, Hereditary spherocytosis, Hemolytic anemia, Whole-exome sequencing

## Abstract

**Background:**

Hereditary spherocytosis and hereditary dRTA are associated with mutations in the *SLC4A1* gene encoding the AE1. In this study, some patients with clinical evidence of congenital HS and renal symptoms were investigated.

**Methods:**

Twelve patients with congenital HS and renal symptoms were recruited from Ali-Asghar Children’s Hospital (Tehran, Iran). A patient suspected of having dRTA was examined using WES method, followed by Sanger sequencing.

**Results:**

One patient (HS03) showed severe failure to thrive, short stature, frequent urinary infection, and weakness. A homozygote (rs571376371 for c.2494C>T; p.Arg832Cys) and a heterozygote (rs377051298 for c.466C>T; p.Arg156Trp) missense variant were identified in the *SLC4A1* and *SPTA1* genes, respectively. The compound heterozygous mutations manifested as idRTA and severe HS in patient HS03.

**Conclusion:**

Our observations, for the first time, revealed clinical and genetic characteristics of idRTA and severe HS in an Iranian patient HS03.

## INTRODUCTION

Hereditary hemolytic anemia is a form of anemia where red blood cells (erythrocytes or RBCs) are destroyed. Types of congenital hemolytic anemia include red cell membrane disorders, RBC enzyme disorders, and hemoglobin production disorders^[^^1^^,^^2^^]^. HS is the most common form of congenital hemolytic anemia, with wide clinical heterogeneity ranging from an asymptomatic situation to severe hemolysis^ [^^3^^]^. HS is primarily diagnosed by anemia, jaundice, and spherocytes in peripheral blood smear, splenomegaly, cholelithiasis, and positive family history^ [^^4^^-^^6^^]^. 

Five genes are associated with spherocytosis, including *SPTA1*, *SPTB*, *ANK1*, *SLC4A1*, and *EPB42*, which are involved in the interplay between the erythrocyte membrane and the lipid bilayer^[^^3^^,^^4^^]^. *SPTA* plays an important role in the deformability and stability of erythrocytes. Mutations in this gene have been associated with hereditary red blood cell disorders, including HS type 3, hereditary pyropoikilocytosis, and elliptocytosis 2^[^^1^^,^^2^^]^. *SLC4A1 *encodes the human AE1 and is expressed both at the red blood cell membrane and the distal tubules of the kidney^[^^3^^]^, but with different promoter regions and alternative splicing. Thus, *SLC4A1* gene mutations can cause either dRTA and/or hemolytic anemia^[^^1^^,^^4^^]^. RTA syndromes are separated into four types, classic distal (Type 1), proximal (Type 2), mixed-type proximal and distal (Type 3), and hyperkalemic distal (Type 4), which are differentiated by the family history, the presenting manifestations, the biochemical profile, and the radiological findings^[^^5^^]^. Primary dRTA, also referred to as type 1 RTA, a rare genetic disorder with genetic heterogeneity, is caused by loss-of-function mutations in genes associated with primary dRTA. Three genes, i.e. ATP6V1B1, ATP6V1B1, and AE1/SLC4A1, are known to give rise to dRTA^[^^4^^,^^6^^]^. Based on Human Gene Mutation Database (http://www.hgmd.org), in these genes, approximately 94 mutations are identifiable in 70%–80% of dRTA patients^[^^4^^,^^7^^]^. dRTA is commonly presented with metabolic acidosis and normal serum, renal stones, failure to thrive, rickets, hypokalemia, hypercalciuria, and nephrocalcinosis in children^[^^6^^,^^8^^,^^9^^]^. Mutations in the *SLC4A1* gene are reported to be mainly associated with either autosomal dominant or AR dRTA, while those of ATP6V1B1 and ATP6V0A4 are mostly in the form of the AR disease^ [^^6^^]^.

In this paper, we report a family with homozygous mutation (rs571376371 for c.2494C>T; p.Arg832Cys) in SLC4A1 gene and also a heterozygous mutation (rs377051298 for c.466C>T; p.Arg156Trp) in *SPTA1* gene. WES technique was used to elucidate the disease-causing mutations in Iranian HS and the inheritance pattern in patients. 

## MATERIALS AND METHODS

**Patients**

Twelve patients with the primary clinical diagnoses of congenital HS, showing symptoms related to kidney problems, were retrospectively examined. The patients were selected from Ali-Asghar Children’s Hospital, Blood Clinic, Iran University of Medical Sciences, Tehran, Iran. Their medical records were reviewed to determine the clinical features, laboratory data, and disease history (jaundice, peripheral blood smears, family history, failure to thrive, rickets, and nephrocalcinosis). A complete blood count with the accurate morphological detection of blood smears and also abdominal ultrasound screening were performed in the mentioned hospital. Each patient was evaluated for clinical and laboratory parameters related to the manifestations of dRTA. HS patients with no renal symptoms were excluded from the investigation.

**Genetic study**

Patient HS03 with the initial manifestation of kidney problems (abnormal urine pH, severe failure to thrive, repeated urinary infection, and weakness) was selected for WES and referred to Dr. Zeinali’s Medical Genetics Lab. (Tehran, Iran) for genetic counseling and genetic diagnosis. Peripheral blood samples (4-10 ml) were drawn from the family and collected on EDTA. DNA extraction was performed from peripheral blood samples using the KBC Blood DNA Extraction Kit (Kawsar Biotech Co., Tehran, Iran, KBC) following the manufacturer’s instruction, and the samples were sent for WES to decode genetics Company (http://www.decode.com). Confirmation of the result was performed using Sanger sequencing. The amplification was performed in a volume of 25 μL, containing 20 μL of Taq PCR Master Mix (KBC), 2 μL of genomic DNA, 0.3 μL(30 pmol) of upstream primer, and 0.3 μL(30 pmol) of downstream primer, and 1 U of Taq DNA polymerase. ddH_2_O was added to reach a total volume of 25 μL. The amplification was performed using a thermal cycler (Eppendorf, Germany) with the following parameters: first initial denaturation step at 95 °C for 5 min, followed by 28 cycles of denaturation at 95 °C for 1 min, annealing at each optimum annealing temperature for 1 min, extension at 72 °C for 1 min, and final step of 10 min at 72 °C. All primers were designed with PerlPrimer soft-ware (http://perlprimer.sourceforge.net/). The primers for exon 2 of *SLC4A1* gene included 5’**-**TT CTGTTCAAGCCACCCAAG**-**3’ and 5’-CCCAGACT TTACCCATGACTC**-**3’ (PCR product size of 410 bp). The primers for exon 4 of SPTA1 were 5’**-** CAAGTCTCTGAGTGTTTCCC-3’ and 5’-CTTGTG AGTAGTCTGCAGTAAT-3’ (PCR product size of 296 bp).

**WES assay and** ***in silico*** **analysis**

Samples were chosen for WES using the TruSeq Nano sample preparation method and sequenced on Illumina's HiSeq X Ten machines (http://www. decode.com). More than 95% of the targeted sequences were covered adequately for high confidence variant calling. Sequencing reads were aligned to NCBI's Build 38 of the human reference sequence, to the hg19/GRCh37 reference genome, using the Burrows-Wheeler Aligner software (version 0.7.10). Alignments were merged into a single BAM file and marked for duplicates using Picard 1.117. Variants and reads were called by the aid of 2014.4-2-g9ad6aa8 of the Genome Analysis Toolkit and multi-sample configuration, respectively. A filtering route was constructed to select candidate variants in coding regions using general population datasets, specific disease mutation datasets, and in-house variant frequency dataset. To predict the possible impact of variant pathogenicity in exons, *in silico* function was predicted by several software, including SIFT, PolyPhen-2, and Align-Grantham Variation Grantham Deviation. To confirm pathogenic mutations, bidirectional Sanger sequencing was performed using BigDye Terminator Kit (Thermo Fisher Scientific Inc. Foster City, CA, USA, TF), and the samples were run on ABI 3130XL Genetic Analyzer in KBC. All results of sequences were evaluated by the NCBI database (http://www.ncbi. nlm.nih.gov/). 

**Ethical statement**

The above-mentioned sampling was approved by the Ethics Committee of Kawsar Human Genetics Research Center (LGRC), Country (ethical code: 14006318). Written informed consents were provided by the patients’ parents.

## RESULTS

**Clinical follow-up and clinical findings**

**Fig. 1:**
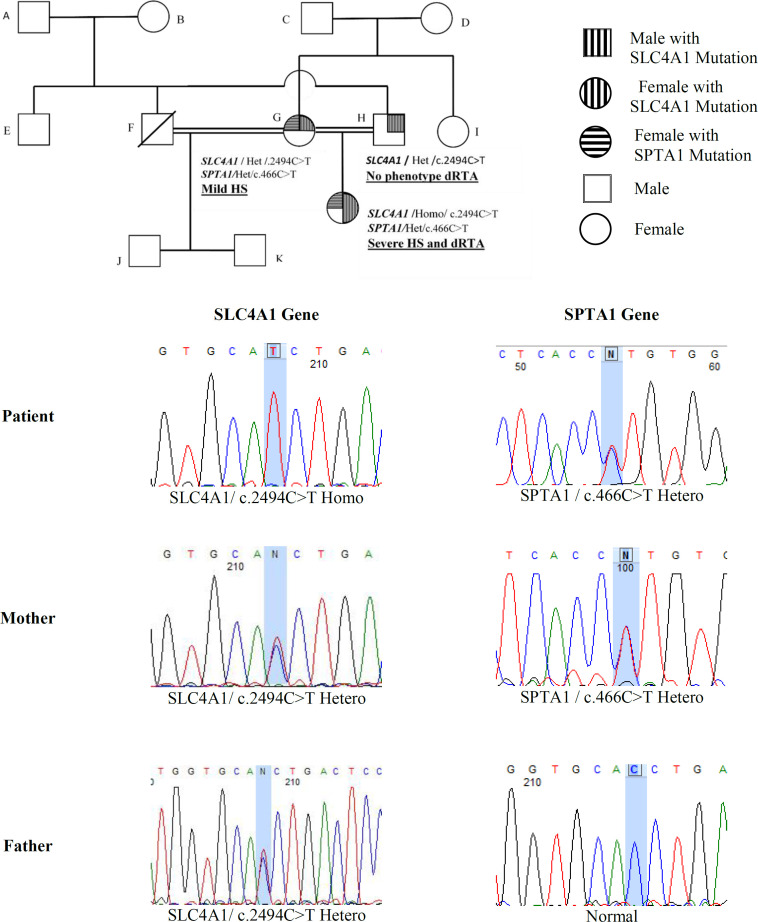
Genotype family pedigree HS03

Twelve samples, having anemia and primary clinical diagnosis of HS, were enrolled in the study. Laboratory findings and clinical characteristics at the time of study are summarized in Table 1. Two patients had consanguineous marriage (patients nos. HS03 and HS05). All patients were identified with failure to thrive and splenomegaly, and no patient was detected to have nephrocalcinosis (Table 1). The patient HS03 had a history of significant symptoms of renal problems. She was a seven-year-old girl with severe jaundice, pallor, anemia, and hemolysis few days after birth and diagnosed with severe HS requiring blood transfusion. Other manifestations include severe failure to thrive, short stature (weight of 19. 5 kg and height of 120 cm), repeated urinary infection, and weakness (Tables 1 and 2). HS03 born to consanguineous marriage with no family history of anemia or renal disease. At birth, the proband had the erythrocyte hemoglobin of 11.6 g/dL, mean corpuscular volume of 84.8 fl, and mean corpuscular hemoglobin concentration of 34.8 g/L. An increase in red cell osmotic fragility was observed, as well. Thalassemia and G-6-PD deficiency were ruled out, and Coombs test was normal. At age 10, the proband was under the supervision of a nephrologist because of failure to thrive and urinary tract infection. Her first dimercaptosuccinic acid renal scan, at age one years, showed left pole damage. Renal ultrasound study after three months indicated no signs of nephrocalcinosis, though few small gallstones were identified. In addition, the proband did not show auditory deficits or cognitive impairment. Her parents had no splenomegaly, or renal failure, or bone disease at the time of study. The patient and her parents were reviewed for clinical features, hematological data, and biochemical features. Clinical and biochemical features and erythrocyte parameters in the family at baseline are shown in Table 2. WES results in the proband and their parents showed a homozygote VUS in *SLC4A1* gene (rs571376371 for c.2494C>T; p.Arg832Cys). We also detected a heterozygote missense VUS in *SPTA1* gene (rs377051298 for c.466C>T; p.Arg156Trp). These variants were confirmed by segregation analysis within the family using direct Sanger sequencing (Tables 2 and 3 and Fig. 1). The sequencing results were compared with the standard sequences at NCBI. Each sample with a questionable sequencing result was amplified by PCR and verified by bidirectional sequencing. According to the Sanger sequencing results, an AR *SLC4A1* (rs571376371 for c.2494C>T; p.Arg832Cys) and an autosomal dominant *SPTA1* (rs377051298 for c.466C>T; p.Arg156Trp) mutations were confirmed in the proband's family. Sanger sequencing also demonstrated two heterozygote missense variants, SLC4A1 (rs571376371 for c.2494C>T; p.Arg832Cys) and *SPTA1* (rs377051298 for c.466C>T; p.Arg156Trp), in her mother, and just one missense variant, *SLC4A1* (rs571376371 for c.2494C>T; p.Arg832Cys), in her father. Figure 1 illustrates the pedigree analysis of the proband with two missense variants.

## DISCUSSION

In this study, clinical manifestations and laboratory findings of 12 patients showing growth retardation and splenomegaly were documented. Two of the patients displayed a significant urinary infection. Also, no patients exhibited hyperchloraemic metabolic acidosis, abnormal urine pH, hypokalemia, and nephrocalcinosis. One of the 12 patients showed severe failure to thrive, short stature, repeated urinary infection, and weakness. Our report describes an Iranian patient with recessive dRTA pattern, including a compound mutations in the *SLC4A1* gene (rs571376371 for c.2494C>T; p.Arg832Cys) and (rs377051298 for c.466C>T; p.Arg156Trp) in the *SPTA1* gene, by using WES and Sanger sequencing method for verification. This pattern has been observed for the first time by our research team.

Band 3 protein (encoded by *SLC4A1*) expedites chloride/bicarbonate exchange in erythrocytes and kidneys. Also, it serves an important role in the stability and morphology of the erythrocyte membrane^[^^10^^]^. The co-existence of the homozygous *SLC4A1* mutations HS are normally lethal in humans due to the lack of band 3 protein since it causes the erythrocytes to be unstable, leading to severe hemolytic anemia. Moreover, it results in a severe impairment of

kidney function^[^^7^^,^^8^^]^. With treatment, renal failure is rare, but dRTA may give rise to the growth retardation and rickets in children or osteomalacia in adults^[^^11^^]^.

**Table 1:** Laboratory findings and clinical characteristics in 12 samples (at presentation)

**Patient** **No.**	**Sex/age (y)**	**Hb** **(g ** **⁄** **dL2)**	**Hct (%)**	**MCHC (% )**	**RDW (%)**	**Consanguinity**	**Spherocytes**	**Splenomegaly**	**Nephrocalcinosis**	**Initial manifestations**	**other**
HS01	F/13	10.5	30.5	34.4	13.2	-	+	++	_	FTT	--------
HS02	F/42	14.4	41.4	34.8	13.5	-	+	+	_	FTT and UI	Underwent splenectomy and cholecystectomy
HS03	F/7	6.4	18.4	34.8	16.8	+	++	+++	-	Severe FTT, short stature, repeated UI, and weakness	-----
HS04	M/18	17.2	49.3	34	14.2	_	+	-	_	FTT	underwent splenectomy and cholecystectomy
HS05	M/2	11.1	30.4	36.5	20.8	+	+	+	_	FTT	Mother of HS05 case underwent splenectomy
HS06	M/12	8.2	24.7	33.2	22.7	-	+	+	_	FTT	
HS07	F/11	13.4	38.9	34.4	10.5	-	+	+	_	FTT	underwent splenectomy and cholecystectomy
HS08	F/12	9.3	29	32.1	23.8	-	++	++	_	FTT and UI	neonatal exchange transfusion
HS09	M/2	9.4	28.5	33	29.6	-	++	+	_	FTT	Mother of HS09 case underwent splenectomy
HS010	F/3	9.2	26.5	34.7	23.8	-	++	+	_	FTT	Father of HS10 case underwent splenectomy and cholecystectomy
HS011	M/11	9.2	26.3	34.9	16.8	-	++	++	-	FTT	
HS012	F/37	12.1	34.7	34.9	11.8	-	+	+	_	FTT	underwent splenectomy and cholecystectomy

MCHC, mean corpuscular hemoglobin concentration; FTT, failure to thrive; UI, urinary infection

**Table 2:** Clinical and laboratory findings and erythrocyte parameters in HS03 family

	**AC**	**M**	**F**	**Reference interval**
**Age (y)**	**5**	**41**	**37**	
Hemoglobin	6/4	11.6	16.3	11- 15 g/dl
Hematocrit	18.4	33.4	45.3	33 – 45 %
MCHC	34.8	34.7	36	32 – 36 g/dl
RDW	16/8	13.8	12.5	11 – 15 %
Reticulocytes (% )	**7**	2.6	1.6	%
Conclusion of RBC Fragility	Increased	Increased	Normal	
Spherocyte	++	+	-	
Coombs (direct)	Negative	Negative	Negative	
Nephrocalcinosis	Negative	Negative	Negative	
Blood pH	7.32	7.38	7.31	
Blood pCO_2 _(mmHg)	43	46	59	
Serum HCO_3 _(meg/I)	22.2	27.2	29.7	
Serum calcium (meg/I)	9.9	9.4	9.3	8.6-10.2 mg/dl
Serum phosphate (meg/I)	5.0	4.1	4.4	2.6-4.5 mg/dl
Serum potassium (meg/I)	3.9	4.0	4.5	3.2-5.3 mEq/l
Serum Creatinine (meg/I)	0.56	0.97	1.1	0.7-1.4 mg/dl
Serum sodium (meg/I)	138	136	137	132-148 mEq/l
Vitamin D total (25OH)	39.3	21.8	15.9	Sufficient: >30 ng/ml
Urine creatinine (random)	16	61	261	mg/dl
Urine calcium (random)	1.5	3.2	19	mg/dl
Calcium.R/creatinine. R	0.094	0.052	0.073	
Urine chloride (random)	10.0	32.0	126	mEq/l
Chloride.R/creatinine. R	0.63	0.52	0.48	
Urine potassium (random)	3.5	17	117.7	
Urine potassium. R/creatinine. R	0.22	0.28	0.45	
Urine sodium (random)	6.9	9.2	15.0	mEq/l
Urine Phosphate (random)	21	50	37	mEq/l
Urine pH	6.5	6.0	6.5	
Urine pH under paraffin	5.5	Negative	5.5	
Deafness/SNHL	Absent	Absent	Absent	
kidney stone	Absent	+	Absent	
AG	+ 17	13	8.3	
Urine anion gap	+ 0.4	13.2	6	
Delta anion gap	2.7	0.31	0.64	

AC, affected child; SNHL, sensorineural hearing loss

**Table 3:** Assessing the pathogenicity of mutations identified using bioinformatics tools and ACMG Standards

**Family ID**	**Gene**	**Position** **(hg38) HGVSc/HGVSp** **(RefSeq transcript)**	**Variant type** **(Exon no./** **Total exon no.)**	***in silico*** ** prediction**	**ACMG classification/** **inheritance**
HS03	SLC4A1	chr17:44251320 NM_000342.3:c.2494C>T NP_000333.1:p.Arg832Cys	Homo Missense variant Exon19/20	SIFT: deleterious PolyPhen: probably damaging Condle: deleterious loF tool: probably damaging GERP: 4.19	VUS, AR
				
	SPTA1	chr1:158681592 NM_003126.2:c.466C>T NP_003117.2:p.Arg156Trp	Hetero Missense variant Exon4/52	SIFT: deleterious PolyPhen: possibly damaging Condle: deleterious loF tool: benign GERP: 3.25	VUS, AD

AD, autosomal dominant, AR, autosomal recessive, VUS, Variant of uncertain significance

There are two hereditary patterns associated with *SLC4A1* that can cause dRTA. The autosomal dominant pattern is seldom associated with blood cell disorders^[^^12^^]^. The AR pattern is commonly associated with blood cell disorders, leading to hemolytic anemia with abnormal red cell morphology, including HS, hereditary stomatocytosis, South East Asian ovalocytosis and hereditary xerocytosis^[^^6^^,^^7^^]^. HS and dRTA have a mutual relationship^[^^1^^,^^10^^,^^13^^]^ because about 20% of HS patients have also heterozygous *SLC4A1* gene mutations^[^^7^^]^. Mutations resulting in both spherocytosis and dRTA diseases are extremely rare in temperate countries^[^^8^^,^^12^^]^. However, compound mutations of dRTA and HS has been reported, showing severe anemia and splenectomy and requiring blood transfusions^[^^9^^,^^13^^-^^16^^]^. The patients displayed severe HS with complete distal renal tubular acidosis or idRTA^[^^7^^,^^17^^]^.

We identified a variant that has not been reported in patients with idRTA disease using the NGS method in Iran. In this study, two variants in two out of five HS-related genes were found in the proband. The first variant identified in *SLC4A1* gene (rs571376371 for c.2494C>T; p.Arg832Cys) was homozygous in the affected case and was inherited from heterozygous parents. The other variant in *SPTA1* gene (rs377051298 for c.466C>T; p.Arg156Trp), heterozygous in the affected case, was inherited from her heterozygous mother. This variant was recognized with bioinformatics tools and ACMG Standards (Table 3).

A single nucleotide change on exon 19 of the *SLC4A1* gene was detected that caused an amino acid substitution, Arginine to Cysteine at position 832. In addition, in the SPTA1 gene, a single nucleotide change on exon 4 was observed, which caused an amino acid substitution, Arginine to Tryptophan at position 156. Unfortunately, no functional studies were performed on SPTA1 to decipher the precise role of this mutation. The heterozygote variant in *SLC4A1* cannot explain this clinical observation as it is also present in the healthy father. We just may consider it as a factor to increase the effect of other variants. The presence of SLC4A1 homozygous mutation in the HS03 is in line with the diagnosis of idRTA with AR inheritance that could have more severe manifestation, due to the co-presence of the other variants in the *SPTA1* gene. Interestingly, the patient’s mother had compound mutations (rs571376371for c.2494C>T; p.Arg832Cys) in the SLC4A1 gene and (rs377051298 for c.466C>T; p.Arg156Trp) in the SPTA1 gene, which were in the form of heterozygosis. She never showed dRTA symptoms. Therefore, the combined effect of the mutations of two genes in heterozygosis in mother seems to be required to cause clinical manifestations in the affected child. Ribeiro *et al.*^[^^14^^]^ first described a child with severe HS and AR dRTA caused by homozygous AE1 V488M (Band 3 Coimbra) with complete dRTA, which had several blood transfusions. Subsequent studies have reported HS and complete dRTA, including E522K /G701D^[^^17^^]^, C479W /G701D, and homozygous A858D/A858D^[^^16^^]^. Other investigations have implied that homozygous *SLC4A1* mutation (Ser667Phe) leads to HS and idRTA^[^^17^^,^^18^^]^. WHS analyzes these exons in a rapid and cost-effective method to recognize new pathogenic genes in rare disorders^[^^4^^,^^11^^]^, especially when family history is uninformative, or when physical examination and routine laboratory findings cannot recognize hemolytic effect and types of inherited hemolytic anemia^[^^19^^,^^20^^]^. Our study shows that NGS is a beneficial diagnostic strategy to facilitate the molecular diagnosis of novel mutations in affected patients of dRTA, especially in recessive forms of dRTA. Potential hematologic and renal complications of dRTA can be quite severe; therefore, timely treatment with bicarbonate supplementation may improve the condition of the patient^[^^4^^,^^11^^]^.

Analysis of the pathogenic mutations of HS is complex because of the phenotypic heterogeneity. This complexity arises from sporadic mutations and the fact that there are no hotspot mutations. According to the reported mutations of pathogenic HS genes, most mutations are autosomal dominant and novel^[^^21^^]^. The present study suggests that NGS test to be used as a rapid screening method for the detection of mutations in hereditary spherocytosis.
